# Population genetic structure and habitat connectivity for jaguar (*Panthera onca*) conservation in Central Belize

**DOI:** 10.1186/s12863-019-0801-5

**Published:** 2019-12-27

**Authors:** Angelica Menchaca, Natalia A. Rossi, Jeremy Froidevaux, Isabela Dias-Freedman, Anthony Caragiulo, Claudia Wultsch, Bart Harmsen, Rebecca Foster, J. Antonio de la Torre, Rodrigo A. Medellin, Salisa Rabinowitz, George Amato

**Affiliations:** 1 School of Biological Sciences, the University of Bristol, Bristol, UK; 2 Sackler Institute for Comparative Genomics, American Museum of Natural History, New York City, USA; 3 The Wildlife Conservation Society, New York City, USA; 4 Columbia University in the City of New York, New York City, USA; 5 Panthera, New York City, USA; 6 City University of New York, New York City, USA; 7 Environmental Research Institute, University of Belize, Belmopan, Belize; 8 Instituto de Ecologia, Universidad Nacional Autonoma de Mexico, Mexico City, Mexico; 9 Southampton University, Southampton, UK; 10 School of Environmental and Geographical Sciences, University of Nottingham Malaysia, Semenyih, Malaysia

**Keywords:** Conservation genetics, Population structure, Jaguar, Felidae, Landscape permeability, Functional habitat connectivity

## Abstract

**Background:**

Connectivity among jaguar (*Panthera onca*) populations will ensure natural gene flow and the long-term survival of the species throughout its range. Jaguar conservation efforts have focused primarily on connecting suitable habitat in a broad-scale. Accelerated habitat reduction, human-wildlife conflict, limited funding, and the complexity of jaguar behaviour have proven challenging to maintain connectivity between populations effectively. Here, we used non-invasive genetic sampling and individual-based conservation genetic analyses to assess genetic diversity and levels of genetic connectivity between individuals in the Cockscomb Basin Wildlife Sanctuary and the Maya Forest Corridor. We used expert knowledge and scientific literature to develop models of landscape permeability based on circuit theory with fine-scale landscape features as ecosystem types, distance to human settlements and roads to predict the most probable jaguar movement across central Belize.

**Results:**

We used 12 highly polymorphic microsatellite loci to identify 50 individual jaguars. We detected high levels of genetic diversity across loci (H_E_ = 0.61, H_O_ = 0.55, and N_A_ = 9.33). Using Bayesian clustering and multivariate models to assess gene flow and genetic structure, we identified one single group of jaguars (K = 1). We identified critical areas for jaguar movement that fall outside the boundaries of current protected areas in central Belize. We detected two main areas of high landscape permeability in a stretch of approximately 18 km between Sittee River Forest Reserve and Manatee Forest Reserve that may increase functional connectivity and facilitate jaguar dispersal from and to Cockscomb Basin Wildlife Sanctuary. Our analysis provides important insights on fine-scale genetic and landscape connectivity of jaguars in central Belize, an area of conservation concern.

**Conclusions:**

The results of our study demonstrate high levels of relatively recent gene flow for jaguars between two study sites in central Belize. Our landscape analysis detected corridors of expected jaguar movement between the Cockscomb Basin Wildlife Sanctuary and the Maya Forest Corridor. We highlight the importance of maintaining already established corridors and consolidating new areas that further promote jaguar movement across suitable habitat beyond the boundaries of currently protected areas. Continued conservation efforts within identified corridors will further maintain and increase genetic connectivity in central Belize.

## Background

The jaguar (*Panthera onca*), listed as near threatened by the IUCN Red List of Threatened, species [1], is impacted by the depletion of wild prey, direct persecution and habitat loss [2]. As small populations become isolated by the fragmentation of habitat, jaguars become more vulnerable to demographic fluctuations, environmental perturbations, and inbreeding depression. Effective conservation planning for the jaguar requires comprehensive studies that effectively link the landscape with genetic structure and gene flow of the species at risk [3, 4]. Knowledge of the demographic history, movement patterns, behaviour and genetic diversity of a species are critical components to make adequate conservation and landscape planning decisions [5, 6].

Jaguars are elusive top predators with large individual home ranges and occurring at low densities in Neotropical forests [7]. After a century of persecution and suffering habitat loss, jaguar range decreased by 48% for the entire Americas and 85% outside Amazonia, [2, 8]. Furthermore, jaguar core areas contracted by 33% in the Gran Chaco [9]. To ensure population viability, the remaining fragmented patches of jaguar habitat need to be connected via a network of corridors that ensure demographic and genetic exchange [6, 10, 11]. Habitat deterioration, human-wildlife conflict and the complex dynamics of jaguar populations are rapidly shaping current patterns of genetic diversity [3, 12, 13].

The establishment of corridors to improve population connectivity, particularly in Mesoamerica, has been among some of the most important efforts to prevent the loss of biodiversity in the world’s biologically richest regions [14]. One of the jaguar populations with the highest density in Mesoamerica can be found in the forests of Belize, a core and critical area for the species throughout its range [15]. However, jaguars in Belize are mainly threatened by overhunting of wild prey, direct persecution by humans, and habitat loss [16, 17].

According to the Belize National Protected Areas System Plan [18], 36% of Belize’s land territory is under conservation management. In particular, with its 514 km^2^ of protected forests, the Cockscomb Basin Wildlife Sanctuary harbours one of the largest concentrations of jaguars in the country [15, 19]. The Cockscomb Basin Wildlife Sanctuary is part of a larger conglomerate of protected areas, the Maya Mountains, forming the largest block of contiguous connected forest in Belize. This area is connected to the northern Selva Maya that extends over Belize to northern Guatemala and southeastern Mexico through a network of forest reserves, ending in an unprotected bottleneck stretch of forest in Central Belize.

The Maya Forest Corridor (formerly the Central Belize Corridor) was established in 2013 by the government of Belize and represents the only remaining source of biological connectivity between the Selva Maya forests in northern Belize and the protected areas of the Maya Mountain Massif in southern Belize [16, 20]. This corridor extends over 750 km^2^ and is comprised of mostly private lands but also communities, and protected areas including: the Labouring Creek Jaguar Corridor Wildlife Sanctuary (LCJCWS), the Peccary Hills National Park, and the Manatee Forest Reserve on national land and private protected areas such as Runaway Creek and Monkey Bay [20]. The Maya Forest Corridor is considered the most critical and important corridor of the Belize National Protected Areas Systems [20]. Within Central America, the Maya Forest Corridor has been considered the corridor with the highest probability of jaguar presence but also with the highest deforestation rate for large-scale agricultural developments, which have resulted in more than 65% reduction of natural habitat over the past decade, and as such in urgent need of protection [21, 22].

Range-wide movement corridors have been established as a major tool to improve population connectivity and thus aid the persistence of the jaguar across its range [23]. Conservation efforts have focused on a broad-scale and would benefit from information gained at a finer scale, especially across heterogeneous or fragmented landscapes [4, 11, 24]. The effective collaboration among scientists, practitioners, non-governmental organisations and politicians will tap the full potential of corridor projects and conservation actions across the jaguar’s range.

Former non-invasive genetic studies on Neotropical felids in Belize confirmed the importance of Selva Maya jaguars and showed that jaguars sampled in Belize and northern Guatemala formed one genetic cluster with high levels of genetic diversity and gene flow between sites [13]. A countrywide non-invasive genetic study focusing on jaguars, pumas (*Puma concolor*) and ocelots (*Leopardus pardalis*) in Belize also detected moderate to high levels of genetic diversity and gene flow for jaguars but also indicated that fine-scale genetic subdivision was present between the most northern and southern sites within the country [25, 26]. The study suggested that human-altered landscapes adjacent to these sites most likely start to negatively impact jaguar movement and gene flow and further recommended continued genetic monitoring of these areas of conservation concern, including central Belize and the Maya Forest Corridor, which represents a critical link between jaguar habitats in northern and southern Belize.

Here, we present a fine-scale conservation genetic study assessing genetic structure and patterns of landscape connectivity for jaguars in central Belize to identify areas at risk. Using non-invasive genetic sampling and faecal DNA, we investigate population genetic structure, levels of genetic diversity, inbreeding and gene flow. Additionally, we compared landscape features to examine landscape permeability for jaguars between the Cockscomb Basin Wildlife Sanctuary and the Maya Forest Corridor in central Belize.

## Results

### Genetic variation

A total of 536 scat samples collected across two areas in central Belize were positively matched to *P. onca*. (*n* = 414 Cockscomb Basin Wildlife Sanctuary; *n* = 126 Maya Forest Corridor). Other identified species included *Puma concolor, Leopardus wiedii, Leopardus pardalis, Herpailurus yagouaroundi,* and *Canis familiaris.* Genotyping revealed a total of 50 unique multilocus genotypes (37 from the Cockscomb Basin Wildlife Sanctuary and 13 from the Maya Forest Corridor); these included 41 prospective males, 3 prospective females and 6 unidentified genders (Additional file 3). MICROCHEKER detected three loci (FCA212, FCA229, and FCA075), showing signs of a null allele, but did not find evidence of scoring mistakes or large allele dropout (Additional file 1).

**Fig. 1:**
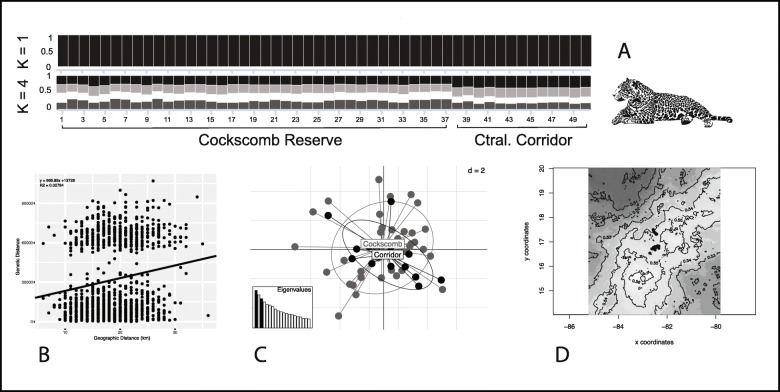
Genetic population structure of jaguars in central Belize. **a** Assignment probabilities of population structure as shown in STRUCTURE for K = 1 and K = 4, each bar represents a single jaguar individual; **b** Mantel’s test for correlation between genetic distance and geographic distance (km) showed low correlation, R_xy_ = 0.167, *P*-value = 0.010 from 10,000 randomizations; **c** Scatter plot of first two principal components (*PCs*) of the DACP analysis of genetic diversity, dots represent pairwise correlations between genetic and geographic distances. Bar plot displays the eigenvalues associated with the components; **d** GENELAND map of population membership probability for the most likely K = 1. Grayscale shading represents the posterior probability of belonging to a single cluster (K = 1). Basemap: DIVA-GIS, Belize map data©2019

Twelve microsatellite loci were successfully amplified with a mean expected heterozygosity HE = 0.61 (SD = 0.042), and a mean observed heterozygosity HO = 0.55 (SD = 0.05). The mean number of alleles per locus (NA = 9.33) ranged from 3 to 10 (Table 1). The mean polymorphic information content PIC = 0.642. We determined the geographical coordinates assigned to each individual by averaging the coordinates of all the samples corresponding to that particular individual. Tests for departure from Hardy-Weinberg Equilibrium followed by Bonferroni correction were variable for each locus, with four loci deviating from HWE (Table 1). This deviation could be explained by a deficit of heterozygotes within the population potentially caused by inbreeding or by the presence of null alleles (FIS = 0.22, *p*-value = 0.001). Furthermore, this test showed evidence of low genetic differentiation (FST = 0.021, *P*-value = 0.007; FIT = 0.237, P-value = 0.001); linkage disequilibrium was not significant for any pair of loci. The DAPC of genetic diversity showed overlapping of the two sites, indicating overlapping of allele frequencies and little differentiation between groups (Fig. 1c). The AMOVA analysis revealed that less than 2% of genetic variance occurred among individuals in the Cockscomb Basin Wildlife Sanctuary and individuals in the Maya Forest Corridor; and showed low levels of genetic differentiation between groups (FST = 0.015, *P*-value = 0.026). Results from the Mantel test showed significant evidence of isolation by distance (Rxy = 0.167, *p*-value = 0.010; Fig. 1b).

**Table 1:** The number of alleles (N_A_), allelic richness (A_R_), observed (H_O_) and expected heterozygosity (H_E_), fixation index (F_IS_), its standard error (SE), and *P*-value for the test of Hardy–Weinberg equilibrium for 12 microsatellite loci amplified for 50 jaguars

Locus	N_A_	AR	H_O_	H_E_	F_IS_	SE	*P*-value
FCA032	5	3.523	0.540	0.629	0.167	0.023	0.219
FCA100	5	3.687	0.520	0.597	0.147	0.056	0.085
FCA124	4	2.989	0.571	0.653	0.119	0.178	0.507
FCA126	6	4.454	0.766	0.709	−0.103	0.035	0.475
FCA212	2	1.989	0.163	0.273	0.437	0.355	**0.015**
FCA229	6	4.172	0.596	0.729	0.240	0.313	**< 0.0001**
FCA096	6	4.565	0.778	0.729	−0.051	0.139	0.584
FCA132	2	1.572	0.085	0.081	0.342	0.005	1.000
FCA275	3	2.995	0.532	0.643	0.217	0.111	**0.010**
FCA075	10	6.852	0.612	0.844	0.261	0.051	**0.001**
FCA208	7	5.533	0.833	0.767	−0.028	0.042	0.926
FCA225	4	3.691	0.524	0.585	0.238	0.024	0.144
	*9.33*		*0.55*	*0.61*	*0.22*		***0.001***

Bold values indicate loci not in Hardy–Weinberg equilibrium (*p* < 0.01) following Bonferroni correction. Estimates across all loci in italics

### Population structure and relatedness

Data analysis using STRUCTURE revealed that K = 1 had the highest mean probability of density value, and K = 4 had the highest delta-K value (Fig. 1a). This was consistent with the results from TESS, where K = 1 also had the highest probability (ΔK = 10.15). In both cases, no clear pattern of genetic structure can be observed when rendering the assignment probability in bar plots (Fig. 1a). Results from GENELAND revealed that K = 1 also had the highest probability in 8 of 10 runs, and the final map does not show a clear population boundary between sampling sites (Fig. 1d). The DAPC analysis showed that the lowest BIC value (68.42) corresponded to K = 2 and is represented in a single discriminant function; however, the BIC difference between K = 2 and K = 1 is negligible (~ 1).

We analysed the performance of seven relatedness estimators to assess the degree of resolution expected in our dataset. Mean relatedness amongst individuals from the Maya Forest Corridor was − 0.046 ± 0.068 (SE =0.008) and from Cockscomb Basin Wildlife Sanctuary − 0.15 ± 0.086 (SE = 0.003). Amongst all individuals mean relatedness was − 0.01 ± SD 0.08 (SE = 0.002). Overall, individuals from the Maya Forest Corridor were more closely related to each other than to those in the Cockscomb Basin Wildlife Sanctuary.

### Landscape permeability

**Fig. 2:**
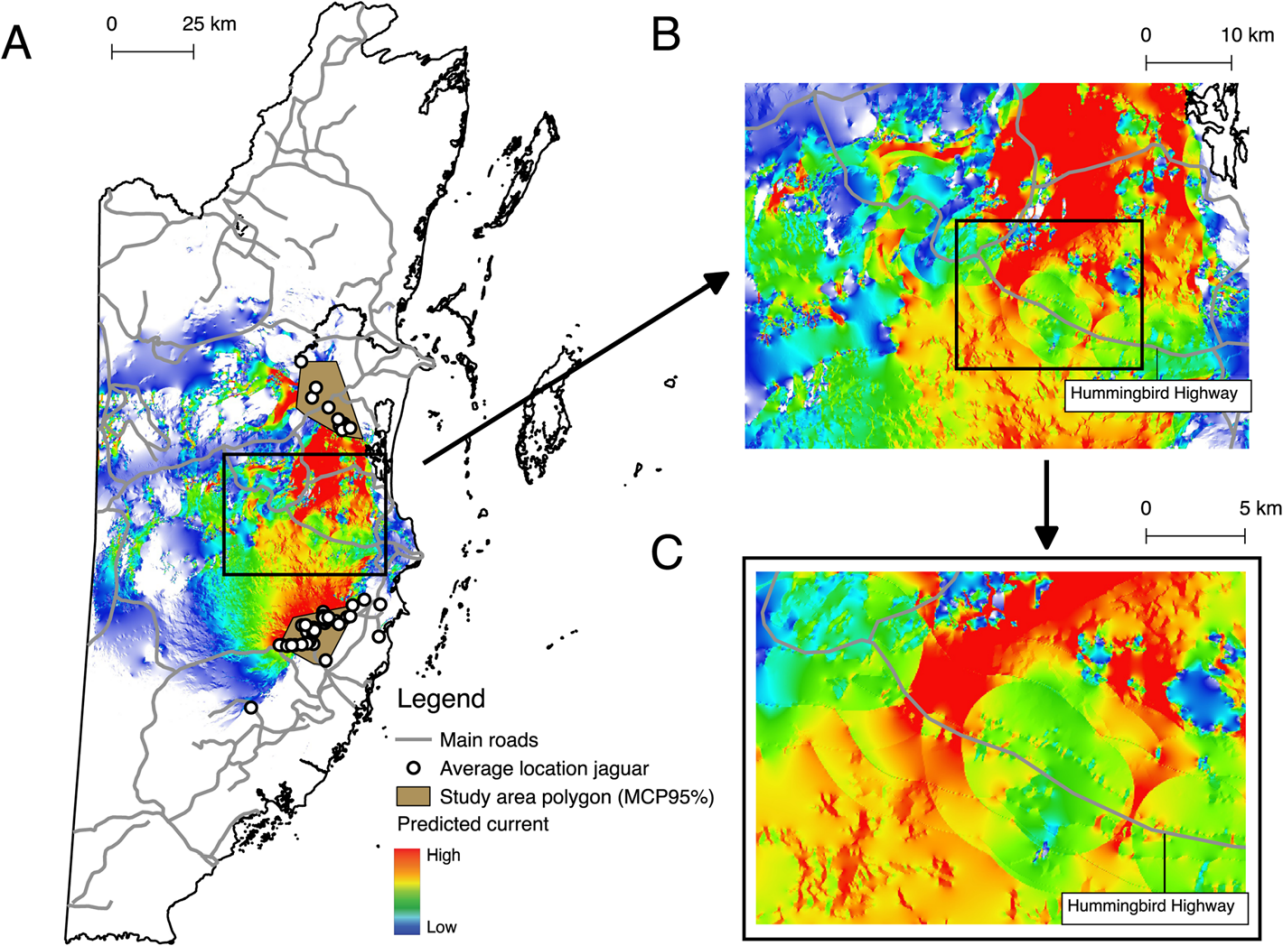
Circuitscape cumulative current maps of the density of potential movement of *P. onca* as a factor of landscape resistance with the effect of habitat preference. **a** Omnidirectional connectivity between the Cockscomb Basin Wildlife Sanctuary and the Maya Forest corridor. **b** Closeup of main areas connecting the two study sites and Hummingbird highway. **c** Closeup for a region of two main pinch points crossing Hummingbird highway. Grey lines denote main roads. White circles denote the average location of individual jaguars identified with the genotype analysis. Brown areas represent the study area as calculated with a 95% minimum convex polygon (MCP95%). The amount of current flow through the landscape ranges from high in red to low in blue. Basemap: DIVA-GIS, Belize map data©2019

Paths of predicted jaguar movement were detected across the two study areas in central Belize (Fig. 2, Additional file 4). We identified important paths that have high potential to facilitate jaguar movement between the Cockscomb Basin Wildlife Sanctuary and the Maya Forest Corridor in central Belize. In particular, we detected two main areas potentially centralizing jaguar movement from Sibun and Sittee River Forest Reserves to Manatee Forest Reserve (− 88.5830 W, 17.0623 N; and − 88.4814 W, 17.0112 N), and vice versa, along a stretch of approximately 18 km adjacent to Hummingbird highway (Fig. 2). The areas connecting one reserve to the other ranges from approximately 25 m to 7 km.

**Fig. 3:**
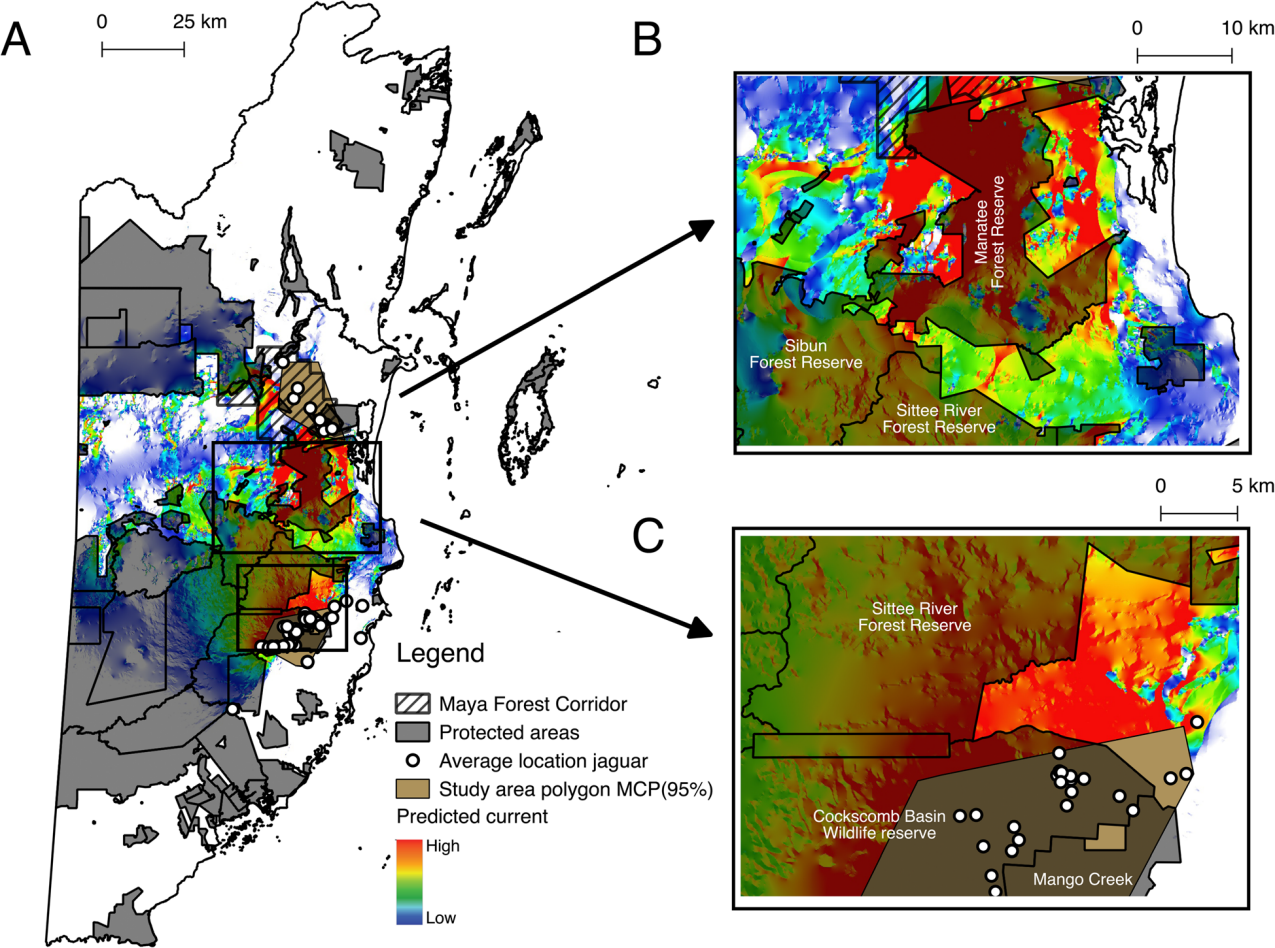
Predicted corridors for jaguar movement between the Cockscomb Basin Wildlife Sanctuary and the Maya Forest corridor that fall outside protected areas in central Belize. **a** Omnidirectional connectivity between the Cockscomb Basin Wildlife Sanctuary and the Maya Forest corridor with protected areas overlapping. **b** Closeup of potential jaguar movement corridors in unprotected areas east, west and south of Manatee Forest Reserve. **c** Closeup of potential jaguar movement corridors in unprotected areas northeast of Cockscomb Basin Wildlife Sanctuary and Mango Creek and east of Sittee River Forest Reserve. Black hatching represents the Maya Forest Corridor. Black-transparent areas represent protected areas. White circles denote the average location of individual jaguars identified with the genotype analysis. Brown areas represent the study area as calculated with a 95% minimum convex polygon (MCP95%). The amount of current flow through the landscape ranges from high in red to low in blue

Circuitscape analysis identified other critical paths of predicted jaguar movement that fall outside the boundaries of the Manatee Forest Reserve and the Maya Forest Corridor. In particular, Circuitscape detected high-currency paths for jaguar movement in approximately 125 km^2^ of unprotected habitat east of Manatee Forest Reserve and 70 km^2^ of unprotected habitat west of the same reserve (Fig. 3, Additional file 4). Furthermore, Circuitscape found paths of high current density for jaguar movement both east and west of Sittee River Forest Reserve and northeast of Cockscomb Basin Wildlife Sanctuary and Mango Creek in an area of approximately 160 km^2^ (Fig. 3).

## Discussion

### Population genetics

This study assessed genetic diversity and structure for jaguars across two study sites in central Belize. Using twelve polymorphic microsatellite loci, we successfully identified 50 jaguar individuals corresponding to 41 males, 3 females and 6 individuals of undetermined sexes. The relatively low number of females could be explained by the sampling methodology rather than reflecting the proportion of sexes in the area. Sampling was primarily conducted along trails, which tend to be dominated by males [27]; because females could be more elusive, have smaller home ranges, hide their scats and avoid crossing open spaces and wide paths [4, 5, 28–30] this method could favour the sampling of male scats and therefore bias the analysis towards the more frequently observed sex. Studies on dispersal in large felines show that males are the dispersing sex, while females tend to be more philopatric [11, 13, 26, 31, 32]; other measurements for genetic differentiation between sexes and more female scat samples are necessary to confirm sex-biased dispersal in this area.

We estimated high levels of genetic diversity and gene flow from data derived from microsatellite analysis and did not detect genetic population structure between the Cockscomb Basin Wildlife Sanctuary and the Maya Forest Corridor (Fig. 1). Overall, our results show evidence of high gene flow between sites with low population differentiation and a lack of heterozygosity within certain loci (Table 1). The results from the Bayesian clustering analyses assigned all 50 jaguar individuals to one single genetic cluster and did not show any clear patterns of population structure (Fig. 1a). Our results are consistent with former studies that revealed jaguar movement across the landscape in Belize and relatively high gene flow [26]. However, the spatial scale of this study and biases towards the sampling of jaguar males could potentially limit some of our results. A small spatial scale relative to the movement of sampled jaguars (mostly males) could make it less likely to detect subtle patterns of genetic population structure. When expanding the geographic scope to the entire country, previous studies revealed fine-scale genetic subdivision of jaguar populations between the most northern and southern sites within the country [25, 26].

In addition, the power of Bayesian clustering to detect population structure has been shown to decrease in accuracy at very low levels of population differentiation [33] as the number of estimated populations can be affected by a violation of model assumptions and cryptic relatedness [34]. Bayesian clustering methods rely on genetic information to ascertain population membership and operate by minimising Hardy-Weinberg and linkage disequilibria [33]. Additionally, properties of our data as sample size, number of loci, polymorphisms, and null alleles could have also influenced their performance [35, 36]. Support for a single population was achieved with GENELAND and DAPC analyses, which indicated a single cluster as the most probable number of populations (Fig. 1c, d). GENELAND assigned individuals to each population considering the sampling locations and measurements of genetic differentiation; as this method considered spatial autocorrelation and is more able to detect low levels of genetic differentiation, it more accurately reflects the true K [37]. The closer relatedness of jaguars from the Maya Forest Corridor than that found for jaguars in the Cockscomb Basin Wildlife Sanctuary could potentially indicate more stable spatial groups or barriers to movement at the Maya Forest Corridor compared to the Cockscomb Basin Wildlife Sanctuary.

The seven-year sampling gap between the collection of scats from the two localities (Cockscomb Basin Wildlife Sanctuary in 2003, and Maya Forest Corridor in 2010) and the sampling bias towards the more dispersing sex (males) could have impacted the results of this study. For instance, our genetic results could reflect dispersal of young males into new territories, hindering possible patters of subtle genetic structuring among females. Limited or sex-biased dispersal between sampling localities could further contribute to genetic differentiation between the localities. Studies conducted with radio telemetry show that jaguars depend on large patches of habitat and can have home ranges that surpass 100 km^2^ [4, 7, 15, 16, 38] however, females have smaller home ranges and tend to avoid roads and human-dominated landscapes at a higher degree, showing preference for intact forests [4, 11, 39]. Although having large home ranges and the ability to move considerable distances, jaguars tend to avoid human-dominated areas and show gender-specific differences [4, 27, 39]. Genetic subdivision due to limited gene flow between jaguar populations has been suggested to be primarily caused by habitat fragmentation, loss and human disturbances [13]. Other studies have also demonstrated that jaguars are highly sensitive to habitat fragmentation in human-dominated landscapes and that although dispersal capabilities of the species may slow the effect of drift, the effect of large-scale habitat loss and fragmentation may contribute to genetic differentiation within a short period of time [3, 6].

### Landscape connectivity

The relatively high levels of gene flow and low genetic differentiation found in our study attest to the successful connectivity between jaguars in the Maya Forest Corridor and the Cockscomb Basin Wildlife Sanctuary, which were a continuum of jaguar habitat in the distant past but that still maintain a single genetic population. Movement corridors are essential to maintain the genetic connectivity for wide-ranging carnivores like jaguars [23, 40] and are an example of an effective conservation strategy that could be implemented in other areas of the species range, such as those in the Atlantic Forest of South America, where there is a lack of genetic and landscape connectivity among isolated remnant jaguar populations [3, 24, 41, 42]. The effect of jaguar corridors on the species conservation is an effective conservation strategy that has also contributed significantly to the conservation and connectivity of a larger number of co-occurring species [40].

Anthropogenic barriers and accelerated rates of habitat conversion into agricultural lands are likely to alter gene flow between core jaguar areas if these are not effectively connected [3, 6, 42, 43]. In this study, we identified several areas in central Belize that are likely to be used by jaguars as they move through the landscape and that represent potential locations for priority management to ensure continued gene flow (Figs. 1, 2). In particular, the negative impact of roads on jaguar populations should be considered to improve existing corridors or in the design of new ones; for example, roads can block the movement of wildlife, and also open up areas to hunters [4, 41, 43].

Using circuit theory, we incorporated landscape features to predict suitable pathways for jaguar movement between the Maya Forest Corridor and the Cockscomb Basin Wildlife Reserve. We pinpointed two main areas of connectivity between Sittee River Forest Reserve and Manatee Forest Reserve along ‘Hummingbird Highway’, a clear anthropogenic boundary between the two sites (Fig. 2). These critical areas are important to maintain connectivity outside the boundaries of Manatee, Sibun and Sittee River Forest Reserves. These results are preliminary and limited by the geographic sampling of our study, in addition to the scarcity of information regarding fine-scale habitat use of jaguars in the area. Furthermore, even though widely utilized, expert knowledge approaches alone are limited and need to incorporate empirical parameterization of the permeability surface to achieve greater accuracy [44–46]. Studies looking at jaguar occurrence, density, behaviour and movement patterns in the region will be critical to creating more accurate connectivity models and improving the reliability of landscape analyses. Studies in central Belize with camera trapping and tracking with GPS radio-collared individuals will confirm the frequency of use of such areas as a passage between reserves and would provide more information on the impact of Hummingbird highway on jaguar movement. The standard means of studying jaguar populations through the deployment of camera traps in Belize [19, 29] can also be augmented with technologies such as three-dimensional remote sensing, which critically improves functional connectivity estimates compared to conventional land cover data [47].

## Conclusion

Our results provide a screenshot of genetic patterns of animals whose scats were sampled during 2003–2011. Our study found high levels of gene flow and the presence of a single jaguar population inhabiting the Maya Forest Corridor and the Cockscomb Basin Wildlife Sanctuary. We identified important areas that have great potential to facilitate jaguar movement and maintain connectivity between the Maya Forest Corridor and the Cockscomb Basin Wildlife Reserve in central Belize (Fig. 2). Our landscape connectivity analysis provides critical information useful to mitigate future effects of habitat fragmentation and anthropogenic activities on jaguar movement and gene flow. The results of this research provide a basis for conservation decisions on jaguars, having to travel through human-dominated landscapes. Non-invasive genetic monitoring has a high potential to provide critical information for conservation planning for threatened wildlife in Belize, as shown in this study. Future studies with large genome-wide datasets will be useful to evaluate fine-scale population structure and will allow investigating loci exhibiting ecologically relevant adaptation [48]. However, in view of the urgent need of understanding genetic variability and population structure, and the relatively ease and reduced cost of using a few loci to understand patterns of genetic diversity, we recommend using the suit of microsatellite markers used in this study (see Additional file 2) to compare jaguar populations in different countries. Furthermore, we recommend sampling in western and southern Belize to better predict habitat connectivity across the Maya Mountain Massif and between this area and the Maya forests in the north. By combining analyses on population genetics, landscape ecology and three-dimensional remote sensing, we will achieve a better understanding of the influence of landscape features and anthropogenic activities on jaguar movement and gene flow across Belize.

The difficulty of studying jaguars in the wild has limited researchers in their efforts to resolve conservation and management issues. Furthermore, given the new environmental challenges and other anthropogenic threats that jaguars and other wildlife face, it is important to support the Belize government to maintain and consolidate corridors that connect suitable habitat and allow the movement of top predators like jaguars. The efforts that international and national conservationists, scientists, local communities and politicians have placed in the last 15 years have helped maintain a relatively healthy jaguar population in central Belize. We encourage continued genetic and ecological monitoring in coordination with management actions taking place in Mexico, Guatemala and other Central American countries to promote connectivity among isolated remnant jaguar populations. Consolidating jaguar corridors and expanding natural protected areas is fundamental to make it a long-term success and one that requires local and international support.

## Methods

### Sampling

Scat collection was conducted by Panthera Belize in two sites (Fig. 2): 1) the Cockscomb Basin Wildlife Sanctuary and some areas that fall beyond the boundaries of this natural reserve, between 2003 and 2007; and 2) the Maya Forest Corridor between 2009 and 2011. Sampling was conducted as part of the Global Felid Genetics Program. The Maya Forest Corridor (17.349140° N, 88.455310° W, 50 m elevation) has been identified as an important link between jaguar populations in northern and southern Belize [20]. The Cockscomb Basin Wildlife Sanctuary (16.7162° N, 88.6608° W, 500 m elevation) supports the highest density of jaguars in Mesoamerica [15, 19]. These two areas play a crucial role in the maintenance of the Mesoamerican Biological Corridor, comprised of a network of protected areas stretching from Mexico to Panama [23]. A total of 852 scats were collected opportunistically along forest trails and unpaved roads. Samples were georeferenced at the time of collection and stored individually at room temperature using silica gel beads until DNA extractions. All faecal material has been deposited in the Sackler Institute for Comparative Genomics at the American Museum of Natural History, NYC.

### DNA extraction and species identification

About 200 mg of the dry sample was shaved from each scat and used to extract genomic DNA using a QIAmp DNA extraction Stool Mini Kit (Qiagen Inc., Valencia, California, USA). Samples were screened to identify species via PCR using three mitochondrial gene regions including cytochrome *b* (H15149) [49, 50], *12S* rDNA (L1085 and H1259) [51] *16S* rDNA (L2513 and H2714) [51] and *16Scp* (16S cp-F 16S cp-R) [51]. PCR amplifications were done using G&E Ready-to-go PCR Beads (GE Healthcare, Piscataway, New Jersey, USA) in 25ul reaction volumes containing 0.2uM of each primer, 0.3uL BSA and 1ul of DNA. PCR profiles for each reaction are available as supplementary material (see Additional file 2). All extractions and amplifications included negative controls. Cycle-sequencing was performed with the BidDye® Terminator v. 1.1 cycle Sequencing kit (Applied Biosystems, Lennik, Belgium) using the PCR primers. Amplified products were visualised and scored in an ABI 3730xl DNA Analyzer (Applied Biosystems, Carlsbad, CA). Sequences were aligned and manually corrected using GENEIOUS v.6.5 (Biomatters Ltd., Auckland, New Zealand). Species identification was confirmed by comparing consensus sequences to known felid references and by constructing a phylogenetic tree with 93% similarity using the Jukes-Cantor neighbourhood-joining model [52]. Species ID was validated if at least three gene fragments successfully identified the same species.

### Individual and sex identification

Samples identified as *P. onca* were genotyped for twelve polymorphic microsatellite loci (FCA 32, 75, 96, 126, 100, 124, 132, 208, 212, 229, 275, and 225) [53]. Each sample was screened 4 times to have enough replicates to assign the alleles correctly and to reduce artefacts caused by allele dropout, very difficult samples were screened up to 6 times. The genotypes were validated if they successfully scored at least three times. Samples that failed to amplify at least six loci were not considered in our analysis. We amplified sets of 2–3 primers in multiplex PCR reactions and FCA225 as singleplex (see Additional file 2). Amplification was performed using the Qiagen Multiplex Master Mix® (Qiagen Inc., Valencia, CA), with a final volume of 20ul following manufacturers recommendations.

PCR reaction conditions were optimised in the annealing temperature for each set of primers as follows: 95 °C for 15 min, followed by 13 cycles of 94 °C for 30s, 57.4–62.4 °C for 90s, 72 °C for 60s, followed by 32 cycles of 94 °C for 30s, 55–60 °C for 90 s, 72 °C for 60s and 60 °C for 30 min (see Additional file 2). Amplified products were visualised and scored in an ABI 3730xl DNA Analyzer (Applied Biosystems, Carlsbad, CA). PCR products were analysed using GeneMapper v5.0 following the quality index measure to validate genotypes (Miquel et al. 2006). In order to further validate the allele calling, we used FRAGMAN v.1.0.8 package in R [54]. Identification of unique multilocus genotypes was achieved using the ALLELEMATCH package in R v.2.5 [55], which accommodates for genotyping error and missing data by making pairwise comparisons and finding similarity scores for each pair of profiles and clustering similar ones. To determine if genotypes were not different individuals matched by chance, the cumulative probability of identity for unrelated individuals (PID) and siblings (Psib) was calculated. Genotypes with Psib < 0.010 were positively identified as single individuals [25, 56].

Gender was determined following the PCR-CTPP method for sex identification developed by Wei et al. (2008) based on zinc finger alleles (ZFX/ZFY). The two forward primers (ZF-1F and ZFY-2F) were fluorescently labelled with FAM and MAX, respectively. Amplification was performed using G&E Ready-to-go PCR Beads (GE Healthcare, Piscataway, New Jersey, USA) in a final volume of 25ul containing 2 mM of each primer and 5ul of DNA. The PCR conditions were as follows: 95 °C for 5 min, followed by 13 cycles of 95 °C for 45 s, 63 °C for 60s, 72 °C for 60s, followed by 35 cycles of 95 °C for 45 s, 53 °C for 45 s, 72 °C for 60s and 72 °C for 10 min (see Additional file 2). DNA amplifications were visualised in a 5% agarose gel to confirm the molecular sexing. Amplified products were visualised and scored in an ABI 3730xl DNA Analyzer (Applied Biosystems, Carlsbad, CA). PCR products were analysed using GeneMapper v5.0.

### Conservation genetics analyses

MICROCHECKER v 2.2.3 [57] was used to screen null alleles at each locus. Measures of genetic diversity as average number of alleles per locus (A), allelic richness (AR), observed (Ho) and expected heterozygosity (He) deviations from Hardy-Weinberg equilibrium (HWE), inbreeding coefficient (FIS), and multivariate analyses were performed using the ADEGENET package in R v.2.0.1 [58]. The rarefaction procedure implemented the HIERFSTAT package in R v 2.9.3.250 [59] was used to estimate the expected number of alleles (N_A_) and to compare allelic richness (A_R_). Genetic differentiation among sampled individuals was summarised in a Discriminant analysis of principal components (DAPC) based on the allele frequencies. Linkage disequilibrium (LD) between pairs of loci was performed in GENEPOP v. 4.2 [60] with default settings. We used GenAlex v6.0 [61] to test the occurrence of a positive correlation (Rxy > 0) between the genetic PHI_PT_ matrix and geographic distance so-called Isolation by Distance (IBD) via Mantel tests of matrix correspondence; we assessed the partitioning of genetic variation between sampling localities with Analysis of Molecular Variance (AMOVA).

### Population structure

We estimated population genetic structure using Bayesian assignment methods with STRUCTURE v2.3.4 [62], which assigns individuals to a number K of genetically homogeneous groups, based on the Bayesian clustering in accordance to the expected Hardy–Weinberg equilibrium and absence of linkage disequilibrium between loci. We ran STRUCTURE using the admixture model with correlated allele frequencies and sampling locations as spatial prior (LOCPRIOR) to allow sampling location to assist in the clustering, and we performed 20 independent runs for K = 1–10. We set a burn-in period of 100,000 and 1,000,000 MCMC iterations and assumed an admixture model with correlated allele frequencies. To determine the optimal number of clusters and render bar plots, we implemented the Evanno method [35] using POPHELPER package in R v1.2.1 [63]. Furthermore, we inferred spatial genetic structure with TESS v2.3.1 [64]. This program assumes that population memberships follow a hidden Markov random field model where the log-probability of an individual belonging to a particular population, given the population membership of its closest neighbours, is equal to the number of neighbours belonging to this population [65]. We tested the CAR, and BYM models with linear trend surface to define the spatial prior for admixture [36]; we set a burn-in period of 100,000 and 1000,000 sweeps through 10 independent runs testing the maximal number of clusters from 1 to 10. To decide the optimal K, we plotted the deviance information criterion (DIC) against K*max*. We also used a spatial clustering approach in GENELAND v4.0.6 [65] as an additional method to infer the number of populations and the spatial location of genetic discontinuities. This program allows using georeferenced individual multilocus genotypes to infer the number of populations and uses the spatial location of genetic discontinuities between those populations. We determined K across 20 independent runs with 1,000,000 MCMC iterations. Thinning was set at 100, allowing K to vary from 1 to 10. We used the correlated allele model and set the maximum rate of the Poisson process at 50 (the number of individuals), the maximum number of nuclei in the Poisson-Voronoi tessellation at 150 (three times the number of individuals), and the uncertainty of spatial coordinates of the collection at 25 m. We re-ran the analysis ten times to check for consistency across runs.

To further explore genetic diversity and structure among individuals, we reduced the dimensions via a Discriminant Principal Component Analysis (DAPC) without a priori group assignment using the ADEGENET package in R v2.0.1 [58, 66]. The tools implemented in DAPC allow solving complex population structures by summarising the genetic differentiation between groups while overlooking within-group variation, therefore achieving the best discrimination of individuals into pre-defined groups [67]. This multivariate method is useful to identify clusters of genetically related individuals when group priors are lacking. Estimation of clusters was performed by comparing the different clustering solutions using the Bayesian Information Criterion (BIC). We compared the results from the three Bayesian approaches and the DAPC to provide confidence in the spatial designation of genetic groupings.

### Relatedness

Levels of genetic relatedness were calculated using seven estimators as implemented in the RELATED package in R v1.0 [68]. Pairwise relatedness was calculated using the estimators described by Queller and Goodnight, 1989 [80]; Li et al., 1993 [81]; Ritland, 1996 [82]; Lynch and Ritland, 1999 [83] and Wang, 2002 [84], as well as the dyadic likelihood estimator described in Milligan, 2003 [85] and the triadic likelihood estimator from Wang, 2007 [72]. Genotyping errors and inbreeding estimations were incorporated into the model, and confidence intervals (95%) were obtained through bootstrapping across loci. Allele frequencies were used to simulate pairs of individuals of known relatedness based on Parent-Offspring, Full siblings, Half siblings and Unrelated individuals.

### Landscape connectivity

We used ArcGIS Desktop v10 to model resistance surfaces of the landscape to jaguar movement and CIRCUITSCAPE v3.5 [69] to identify the most probable routes for dispersal and gene flow between the Cockscomb Basin Wildlife Sanctuary and the Maya Forest Corridor by. Our analysis describes the connectivity and permeability of the landscape between the Cockscomb Basin Wildlife Sanctuary and the Maya Forest and was used to predict all possible paths connecting these two areas. We created a resistance raster surface at a 50 m resolution by combining data on ecosystem type, human settlement and roads. Spatial data were obtained from the Biodiversity and Environmental Resource Data System of Belize (BERDS) and included ecosystem types (2017), protected areas (2015), roads (2013), and human settlements (2014) [70].

The probability of jaguar occurrence has been positively associated with forest cover and negatively associated with human activities [71]. We assumed that landscape features vary in their importance to promote connectivity for jaguars; for example, lowland broad-leaved forests may promote jaguar movement while human settlement may limit it. To represent the variation in importance for connecting different areas, we created a surface of cost values of habitat preference (Table 2) based on published scientific literature and expert knowledge of nine jaguar scientists with experience working in Belize and South-Eastern Mexico. Although basing cost values on expert knowledge as quantitative information is controversial, it has been commonly used as a surrogate when empirical data is limited or unavailable [8, 69–72, 86–89]. To gather the information, we asked jaguar scientists to rank habitat preference according to their empirical and theoretical knowledge on habitat preference and resistance features to allow movement of jaguars across the landscape. The expert’s knowledge was supplemented with empirical data of jaguar habitat use [4, 16, 23, 39, 73–79]. Therefore, by expert knowledge, we mean the experts’ personal judgement that agrees with empirical data.

**Table 2:** Cost-values of jaguar habitat preference based on expert knowledge. Ecosystem names follow UNESCO’s classification system. Values range from 1 (not preferred) to 9 (highly preferred)

Landscape feature	Preference
Lowland broad-leaved wet forest	9
Submontane broad-leaved wet forest	8.8
Lowland broad-leaved moist forest	8.7
Submontane broad-leaved moist forest	8.7
Lowland broad-leaved moist scrub forest	7.4
Lowland broad-leaved wet forest: Steep	7.1
Submontane broad-leaved wet forest: Steep	7
Lowland broad-leaved dry forest	7
Submontane broad-leaved moist forest: Steep	6.9
Lowland broad-leaved moist forest: Steep	6.9
Lowland broad-leaved moist scrub forest: Steep	6.1
Mangrove and littoral forest	5.8
Lowland pine forest	5.7
Wetland	5.4
Submontane pine forest	5.4
Water	5.3
Shrubland	5.1
Lowland savanna	4.4
Submontane pine forest: Steep	3.9
*Anthropogenic feature*	
> 5 km distance from Highways	9
> 5 km distance from human settlements	9
> 3 and ≤ 5 km distance from Highways	6.5
> 3 and ≤ 5 km distance from human settlements	6.5
≤ 3 km distance from Highways	6.1
≤ 3 km distance from human settlements	5.4
Highway	3.3
Agricultural uses	2.7
Aquaculture	2
Wasteland	1.8
Urban	1

Each pixel was assigned a cost value in a scale of 1–9, which represents the relative effort required to move from one point to another (higher values facilitate movement) (Table 2). The average locations of the identified jaguars were grouped in single polygons per study area and were calculated as the 95% minimum convex polygon (MCP). The resulting connectivity between polygons is represented as a cumulative current flow map where areas with higher predicted movement are coloured in red and areas of lower predicted movement in blue. The cumulative current maps represent all potential routes that an animal moving from one polygon could use to move across central Belize and potentially disperse to and from the Cockscomb Basin Wildlife Sanctuary to the Maya Forest Corridor (Fig. 2).

## Supplementary information

**Additional file 1: Figure S1.** Probability graphs of K as calculated by Evanno et al. (2005). **Figure S2.** Box plots, and density plot of relatedness values for simulated pairs of individuals of known relatedness. **Figure S3.** Map of ecosystem types in Belize (2017). **Table S2.** Results from MICROCHECKER for 12 microsatellite loci.

**Additional file 2: Table S1.** Excel template with PCR profiles for species identification, microsatellite multiplexes and sex identification.

**Additional file 3: Table S3.** List of genotypes for 50 individual jaguars identified in the Cockscomb Basin Wildlife Sanctuary and the Maya Forest Corridor.

**Additional file 4. **TIF file to be open in GIS with cumulative current map projections of potential movement of *P. onca* between the Cockscomb Basin Wildlife Sanctuary and the Maya Forest Corridor, Belize.

## Data Availability

All data generated or analysed during this study are included in this published article [and its supplementary information files].

## Abbreviations

AMOVA: Analysis of Molecular Variance
BERDS: Biodiversity and Environmental Resource Data System of Belize
BIC: Bayesian information criterion
DAPC: Discriminant analysis of principal components
DNA: Deoxyribonucleic acid
IBD: Isolation by Distance
LCJCWS: Labouring Creek Jaguar Corridor Wildlife Sanctuary
LD: Linkage disequilibrium
MCP: Minimum convex polygon
*P. onca*: *Panthera onca*
PC: Principal component
PCR: Polymerase chain reaction
rDNA: Recombinant DNA
