# Supplementary material for: Population genetic structure and habitat connectivity for jaguar (Panthera onca) conservation in Central Belize
Source: BMC Genet. 2019 Dec 27;20:100. doi: 10.1186/s12863-019-0801-5 (PMC6933898; doi:10.1186/s12863-019-0801-5)
Supplement: Supplementary file 1 — Additional file 1: Figure S1. Probability graphs of K as calculated by Evanno et al. (2005). Figure S2. Box plots, and density plot of relatedness values for simulated pairs of individuals of known relatedness. Figure S3. Map of ecosystem types in Belize (2017). Table S2. Results from MICROCHECKER for 12 microsatellite loci. [file 12863_2019_801_MOESM1_ESM.docx]

**Supplementary Material**


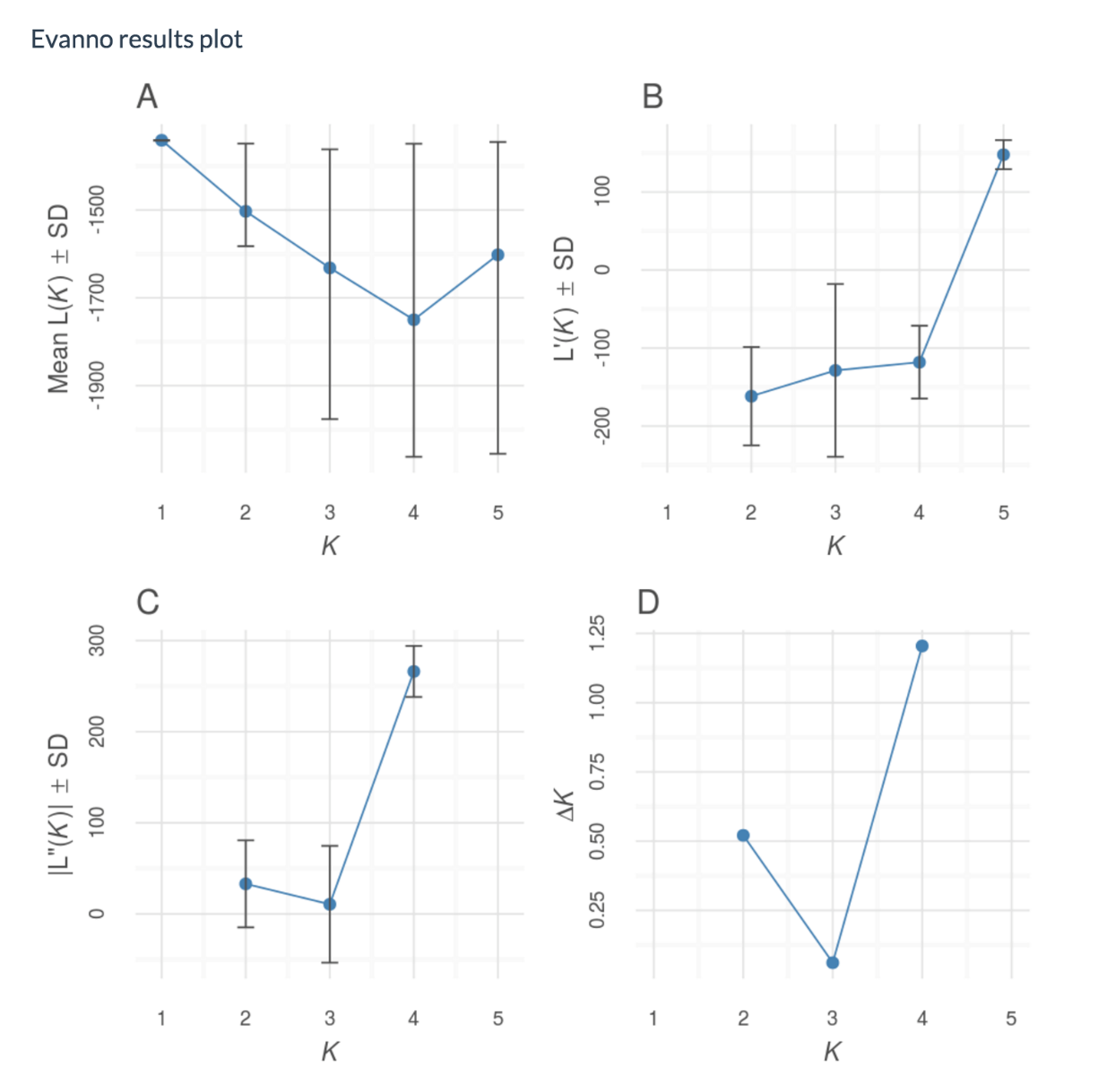


Figure S1. Probability graphs of *K* as calculated by Evanno et al.2005. The plots show the Evanno analysis with (A) estimated log probability of data of runs over increasing values of K, (B) first derivative, (C) second derivative and (D) DK over values of K.

a) b)


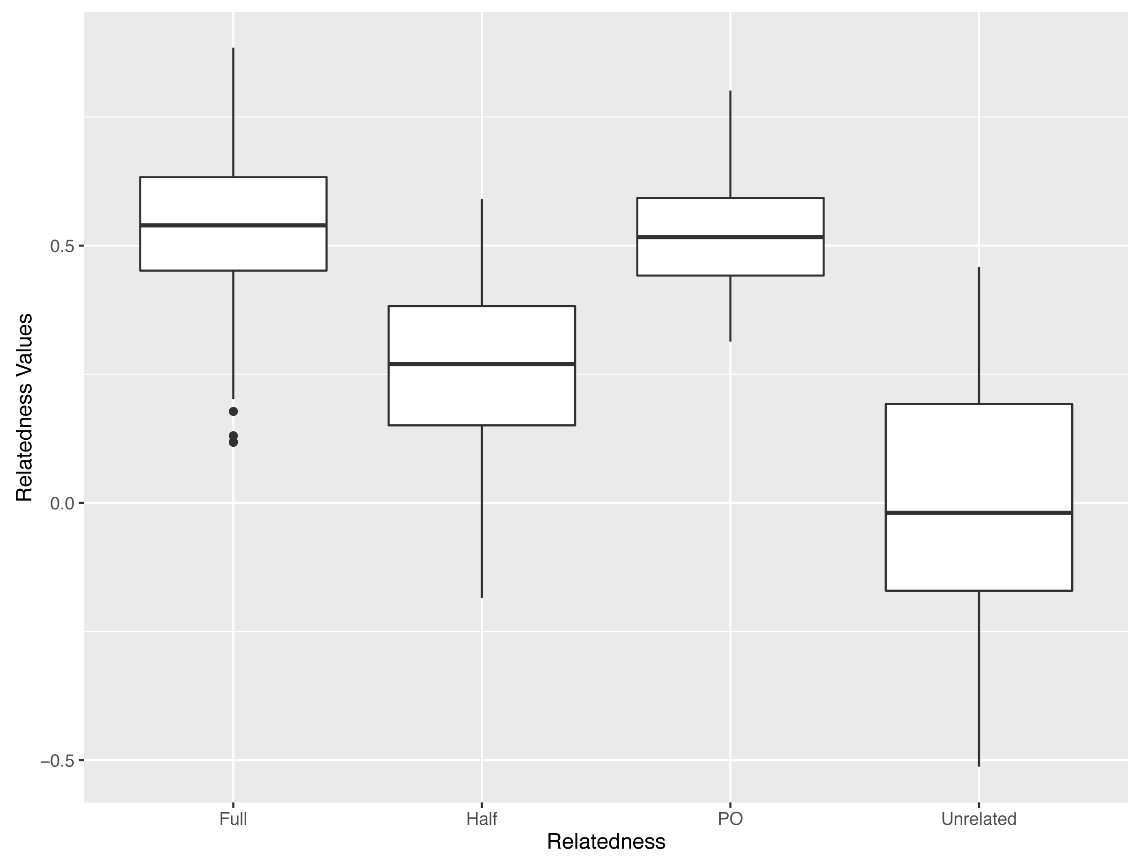

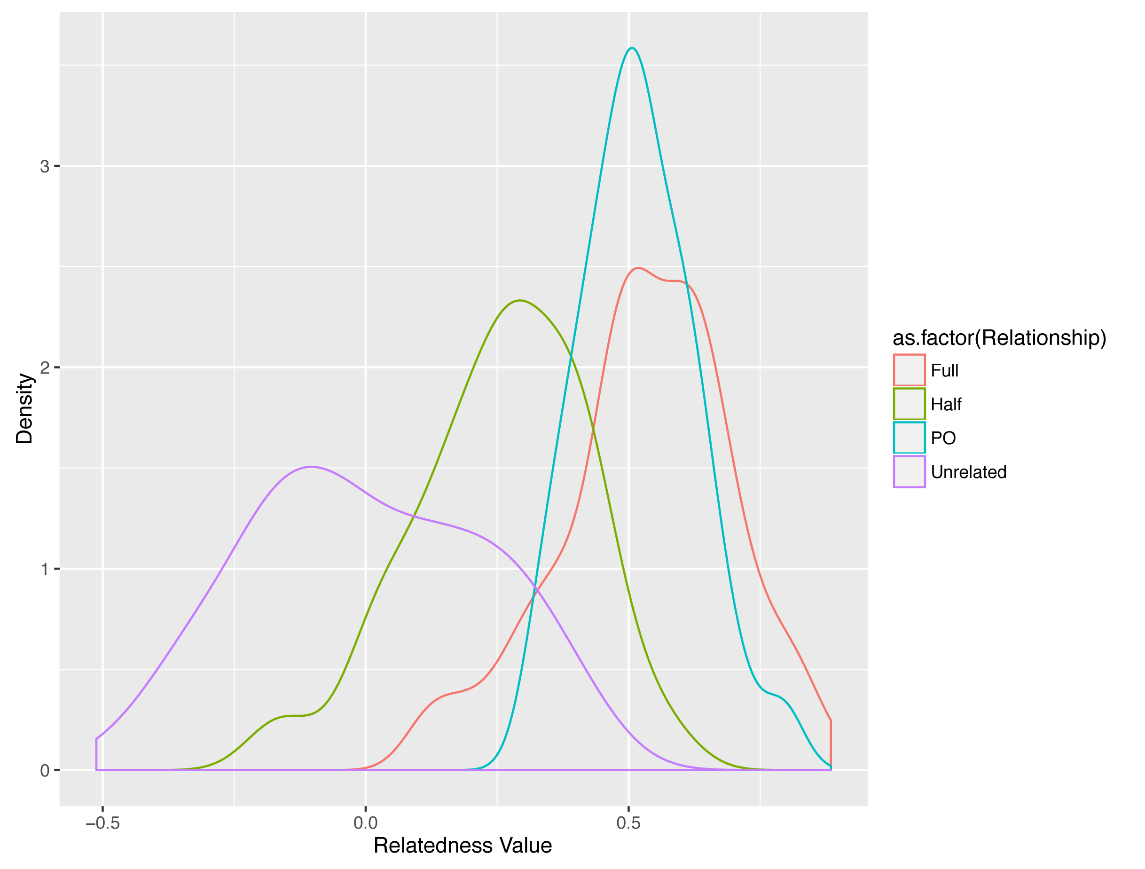


Figure S2. (a) Box plot, and (b) density plot of relatedness values for simulated pairs of individuals of known relatedness. Full siblings (Full); Half siblings (Half); Parent-Offspring (PO); and Unrelated. Based on the estimator of Wang (2002).


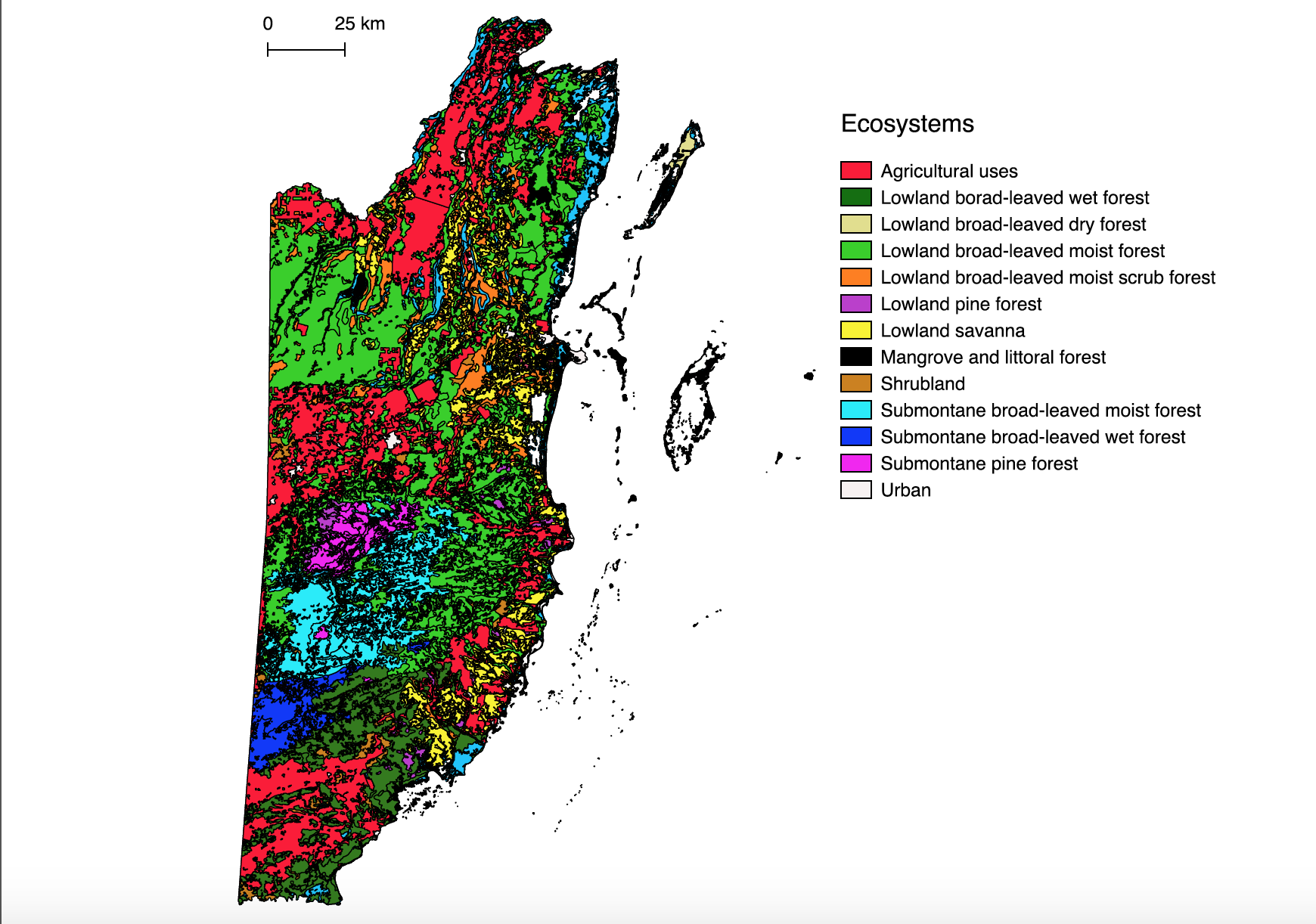


Figure S3. Map of ecosystem types in Belize according to the Biodiversity and Environmental Resource Data System of Belize (BERDS, 2017).

Table S2. Results from MICROCHECKER. This population is possibly in Hardy Weinberg equilibrium with loci FCA212, FCA229, FCA075, showing signs of a null allele. For all loci with null alleles: No evidence for scoring error due to stuttering and no evidence for large allele dropout.

| Locus | Null Present | Oosterhout | | Chakraborty | | Brookfield 1 | | Brookfield 2 | |
| --- | --- | --- | --- | --- | --- | --- | --- | --- | --- |
|  |  |  |  | |  | |  | |  |
| FCA032 | no | 0.073 | 0.076 | | 0.055 | | 0.055 | |  |
| FCA100 | no | 0.056 | 0.069 | | 0.049 | | 0.049 | |  |
| FCA124 | no | 0.066 | 0.067 | | 0.049 | | 0.113 | |  |
| FCA126 | no | -0.063 | -0.039 | | -0.033 | | 0.125 | |  |
| FCA212 | yes | 0.155 | 0.252 | | 0.086 | | 0.168 | |  |
| FCA229 | yes | 0.093 | 0.101 | | 0.077 | | 0.201 | |  |
| FCA096 | no | -0.040 | -0.032 | | -0.028 | | 0.182 | |  |
| FCA132 | no | -0.044 | -0.022 | | -0.003 | | 0.232 | |  |
| FCA275 | no | 0.080 | 0.094 | | 0.068 | | 0.204 | |  |
| FCA075 | yes | 0.138 | 0.159 | | 0.126 | | 0.166 | |  |
| FCA208 | no | -0.046 | -0.041 | | -0.037 | | 0.241 | |  |
| FCA225 | no | 0.037 | 0.055 | | 0.039 | | 0.331 | |  |
